# Catheter-related *Candida*bloodstream infection in intensive care unit patients: a subgroup analysis of the China-SCAN study

**DOI:** 10.1186/s12879-014-0594-0

**Published:** 2014-11-13

**Authors:** Bo Hu, Zhaohui Du, Yan Kang, Bin Zang, Wei Cui, Bingyu Qin, Qiang Fang, Haibo Qiu, Jianguo Li

**Affiliations:** Department of Intensive Care Unit, Zhongnan Hospital of Wuhan University, Wuhan, Hubei, 430071 China; Department of Intensive Care Unit, West China Hospital, Sichuan University, Chengdu, China; Department of Intensive Care Unit, Shengjing Hospital, affiliated to China Medical University, Shenyang, China; Department of Intensive Care Unit, The 2nd Affiliated Hospital of Zhejiang University School of Medicine, Hangzhou, China; Department of Intensive Care Unit, Henan Provincial People's Hospital, Zhengzhou, China; The First Affiliated Hospital of Medical School of Zhejiang University, Hangzhou, China; Department of Intensive Care Unit, Nanjing Zhong-da Hospital, Southeast University School of Medicine, Nanjing, China

**Keywords:** Catheter related infection, Candidemia, Candida parapsilosis, Candida albicans

## Abstract

**Background:**

In patients hospitalized in intensive care units (ICU), *Candida* infections are associated with increased morbidity, mortality and costs. However, previous studies reported confused risk factors for catheter-related *Candida* bloodstream infection (CRCBSI). The objective was to describe the risk factors, microbiology, management and outcomes of CRCBSI in the China-SCAN population.

**Methods:**

Patients with ≥1 *Candida*-positive peripheral blood culture were selected from the China-SCAN study. Peripheral and catheter blood samples were collected for *Candida* isolation. Patients with the same strain of *Candida* in peripheral and catheter blood samples were considered as being with CRCBSI, while patients with *Candida*-positive peripheral blood cultures only or different strains were considered as non-CRCBSI. Data were collected from the China-SCAN study.

**Results:**

CRCBSI incidence in ICU was 0.03% (29/96,060), accounting for 9.86% of all candidemia observed in ICU (29/294). The proportion of CRCBSI due to *Candida parapsilosis* reached 33.3%, more than that of *Candida albicans* (28.6%). In univariate analyses, older age (*P* = 0.028) and lower body weight (*P* = 0.037) were associated with CRCBSI. Multivariate analysis showed that the sequential organ failure assessment (SOFA) score was independently associated with CRCBSI (odds ratio (OR) = 1.142, 95% confidence interval = 1.049-1.244, *P* = 0.002). Catheter removal and immune enhancement therapy were often used for CRCBSI treatment.

**Conclusions:**

In China, CRCBSI was more likely to occur in old patients with low body weight. SOFA score was independently associated with CRCBSI. *Candida parapsilosis* accounted for a high proportion of CRCBSI, but the difference from non-CRCBSI was not significant.

**Electronic supplementary material:**

The online version of this article (doi:10.1186/s12879-014-0594-0) contains supplementary material, which is available to authorized users.

## Background

*Candida sp.* represent the third most common family of pathogens causing bloodstream infections in intensive care units (ICU) patients in the United States [1]-[3]. The global incidence of candidemia is reported to be 6.7-54 per 1000 ICU patients [4]-[6]. Untreated candidemia typically results in eye lesions, skin lesions and abscesses, and often lead to multiple organ failure. The mortality rate is 30-61.8% in Europe and America [5]-[8]. In addition, candidemia can extend hospital stay by 10-30 days, and increase inpatient hospital costs by about $40,000 in the United States [8]. Candidemia requires treatment with an antifungal agent, and removal of the catheter alone is not an adequate therapy for candidemia [9]. The large prospective China Survey of Candidiasis (China-SCAN) study showed that most candidemia in China were caused by non-*albicans* species (58.2%), and that first-line antifungal therapy decreased mortality [10].

Catheters are commonly used in ICU patients, and represent an easy entry route for pathogens, including *Candida sp*. In general, patients with candidemia are inserted with catheters, most commonly central venous catheter (CVC), with a placement rate of 80-89% in Europe and America [11],[12]. CVC placement can significantly increase the risk of candidemia in hospitalized patients [13], and is an independent risk factor for candidemia in the United States [14]. Candidemia caused by catheter placement is named *Candida* catheter-related bloodstream infection (CRCBSI). In addition to CVC, studies in Europe and America identified a number of risk factors that are associated with CRCBSI such as surgical trauma, cancer, parenteral nutrition, diabetes mellitus, urinary catheter, age, vancomycin use, and impaired acute physiology and chronic health evaluation (APACHE) score [8],[15]-[19].

The epidemiology of candidemia varies with geography, but is mostly dominated by *Candida albicans*; however, the proportion of non-*albicans* candidemia is increasing each year [20], sometimes reaching higher rates than that of *albicans* candidemia in European countries [21]. In many countries, *Candida parapsilosis* contributes to 15-20% of candidemia, and is often associated with CRCBSI [22],[23]. Therefore, a better understanding of the CRCBSI epidemiology could lead to better first-line treatments, and to decreased morbidity and mortality.

The China-SCAN study assessed the epidemiology, microbiology, management and outcomes of invasive candidiasis in 67 ICUs across China, and the results were published [10]. The aim of the present study was to assess the risk factors, microbiology, management and outcomes of CRCBSI in the China-SCAN sample. Results might lead to a better identification of patients at high risk of CRCBSI, and to adopt appropriate clinical strategies.

## Methods

### Study design and patients

**Figure 1:**
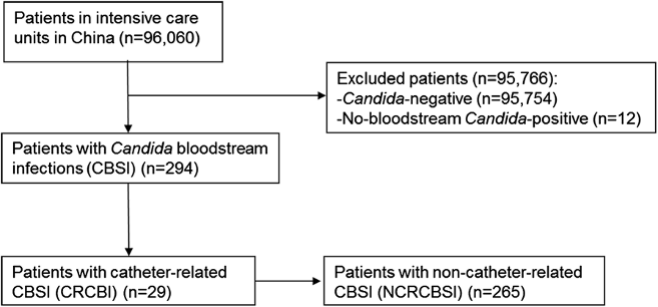
**Flow chart of patients.**

The methods of the China-SCAN study including inclusion and exclusion criteria were previously published [10]. The present study focused on patients with at least one *Candida*-positive peripheral blood culture rather than those with positive *Candida* in histopathological specimen or sterile body cavities fluid specimen culture. Hence, from 306 patients recruited in the CHINA-SCAN study, 294 patients with *Candida*-positive peripheral blood cultures (290 cases with *Candida*-positive peripheral blood culture only, and 4 cases with *Candida*-positive peripheral blood and sterile body cavities fluid specimens) were selected for the present study. Peripheral blood and catheter blood were sampled simultaneously to isolate the *Candida* strains; the same isolated *Candida* strain denoted CRCBSI [21],[24]. Patients with *Candida*-positive peripheral blood culture only or with different *Candida* isolates were considered as non-CRCBSI (NCRCBSI) (Figure 1).

The study was approved by the Ethics Committee of Zhongda Hospital of Southeast University, the lead investigation site. Other participating hospitals accepted the central ethics committee review or conducted a further, independent, ethics review, according to their own institutional policy (20 hospitals). The study complied with the Declaration of Helsinki regarding ethical principles of human subjects research and the relevant ethical requirements of the International Conference on Harmonisation/Good Clinical Practice guidance and national regulations. All patients provided written informed consent. The China-SCAN study is registered with ClinicalTrials.gov (NCTT01253954).

### Sample management and strain identification

All participating centers used the same transportation method. Personnel from each center were trained together to standardize our procedures. Peripheral and catheter blood samples were sampled using strict aseptic methods, and then each sample collected in aerobic and anaerobic blood culture vials (10 ml each). Interval between peripheral and catheter blood sampling was no more than 5 minutes. Blood samples were immediately sent to the sub-center's laboratory for preliminary screening. After preliminary screening, strains were grown in SDA or PDA plastic tubes at 28-30°C and cultured for 2-3 days. After growth, strains were stored at room temperature. Within 1-2 months, strains were transported to the central laboratory. Clinical research assistants at each sub-center were responsible for the regular collection of strains. Strains were shipped to the central laboratory (Research Center for Medical Mycology, Peking University First Hospital, Beijing, China) overnight, at room temperature, by professional courier companies in leak-proof, sealed, unbreakable containers. At the central laboratory, the exact strain and its drug sensibility were determined, as previously described for the China-SCAN study [10],[25].

Strains were identified as previously described for the China-SCAN study [10],[25]. Briefly, species were identified using chromogenic culture media (CHROMagar, Paris, France) and the API 20C AUX yeast identification kit (bioMérieux SA, Marcy l’Étoile, France). When necessary, large-subunit (26S) ribosomal rRNA gene D1/D2 domain sequencing was performed. *Candida haemulonii*, *Candida pelliculosa*, *Candida ernobii*, *Candida norvegensis*, *Candida metapsilosis* and *Lodderomyces elongisporus* were identified by sequencing.

### Data collection and management

All data were from the databases of the China-SCAN study. The content and management of the database had been reported in details [10]. Data were collected from case report forms, and analyzed for comparison between CRCBSI and NCRCBSI.

The APACHE II was evaluated within 24 h of ICU admission. An integrated score from 0 to 71 was calculated. Higher scores indicate more severe disease and higher risk of death [26].

The SOFA score is an ICU scoring system based on organ function or failure rate. The score is based on scores for the respiratory, cardiovascular, hepatic, coagulation, renal and neurological systems [27]. This score was calculated within 24 h of ICU admission.

Chronic hepatic insufficiency was defined as in APACHE II: 1) biopsy-proven cirrhosis and documented portal hypertension; 2) history of upper gastrointestinal bleeding attributed to portal hypertension; or 3) history of hepatic failure/encephalopathy/coma.

### Statistical analysis

Statistical analysis was conducted using SAS 9.1 (SAS Institute, Cary, NC, USA). Continuous variables are presented as means ± standard deviation (SD) (normally distributed), or median (Q1, Q3) (non-normally distributed). Categorical variables are presented as frequencies and percentiles. Independent samples *t*-tests and Wilcoxon rank sum tests were used for continuous variables, as appropriate. Chi-square tests and Fisher's tests were used for categorical data, as appropriate. Variables with *P*-values <0.20 in univariate analyses were entered into a multivariate logistic regression analysis to identify factors that were independently associated with CRCBSI. *P*-values <0.05 were considered to be statistically significant.

## Results and discussion

### Incidence and patient characteristics

Among the 294 identified patients, there were 265 cases of NCRCBSI and 29 cases of CRCBSI. Patients with CRCBSI represented 9.86% of all candidemia patients. Based on the 96,060 ICU patients in the CHINA-SCAN study [10], CRCBSI incidence was 0.03% of ICU patients (29/96,060) (Figure 1).

The baseline characteristics of patients are compared in Table 1. Results from univariate analyses suggest that CRCBSI was associated with age (69.4 ± 19.1 *vs.* 60.7 ± 20.2 years, *P* = 0.028) and with body weight (58.0 ± 5.2 *vs.* 63.2 ± 11.0 kg, *P* = 0.037). SOFA score at candidemia diagnosis (*P* = 0.15), solid tumors (*P* = 0.18) and chronic hepatic insufficiency (*P* = 0.06) were also included in the multivariate analysis.

**Table 1:** **Baseline characteristics of 294 patients with** ***Candida*** **bloodstream infection in the CHINA-SCAN study, according to CRCBSI and NCRCBSI**

Variables	CRCBSI	NCRCBSI	***P***-value
	N = 29	N = 265	
Age (years), mean ± SD	69.4 ± 19.1	60.7 ± 20.2	0.028*
Gender, n (%)			0.527
Male	22 (75.9)	181 (68.3)	
Female	7 (24.1)	84 (31.7)	
Body weight (kg), mean ± SD	58.0 ± 5.2	63.2 ± 11.0	0.037*
Symptoms, n (%)			
Fever	27 (93.1)	243 (91.7)	1.000
Shivers	8 (27.6)	83 (31.3)	0.833
Confusion	13 (44.8)	123 (46.4)	1.000
Concomitant disease, n (%)			
Type 1 or 2 diabetes	7 (24.1)	59 (22.3)	0.833
Chronic cardiac dysfunction	6 (20.7)	57 (21.5)	0.891
Solid tumor	8 (27.6)	45 (16.9)	0.180
Chronic obstructive pulmonary disease	4 (13.8)	31 (11.7)	0.762
Chronic renal insufficiency	5 (17.2)	27 (10.2)	0.259
Chronic hepatic insufficiency	4 (13.8)	12 (4.5)	0.060
Hematological malignancy	0 (0.0)	3 (1.2)	1.000
Invasive procedures within 2 weeks prior to diagnosis, n (%)			
Hemodialysis	2 (6.9)	15 (5.7)	0.259
Invasive mechanical ventilation	24 (82.7)	204 (77.0)	0.666
Total parenteral nutrition	14 (48.3)	115 (43.4)	0.695
Surgery	11 (37.9)	102 (38.5)	1.000
Immunosuppression	2 (6.9)	15 (5.7)	0.679
Illness severity at ICU admission, mean ± SD			
APACHE II score	28.5 ± 7.6	27.0 ± 7.1	0.286
SOFA score	10.6 ± 2.9	11.2 ± 3.5	0.330
Illness severity at diagnosis, mean ± SD			
APACHE II score	28.2 ± 7.2	27.0 ± 7.0	0.360
SOFA score	9.8 ± 3.3	10.8 ± 3.5	0.147
Immune enhancement therapy, n (%)^a^	21 (72.4)	102 (38.5)	<0.001**
Antibiotic use, n (%)			0.963
Monotherapy	8 (32.0)	75 (35.9)	
Two-drug combinations	13 (52.0)	98 (46.9)	
Three-drug combinations	4 (16.0)	35 (16.7)	
Antibiotic use period, mean ± SD	11.4 ± 4.2	10.6 ± 6.5	0.514
Antibiotic therapy >5 days, n (%)	25 (86.2)	209 (78.9)	0.469
Antifungal therapy, n (%)	28 (96.6%)	229 (86.4%)	0.118
Initial antifungal treatment, n (%)			0.977
Fluconazole	11 (39.3%)	84 (36.7%)	
Caspofungin	7 (25.0%)	54 (23.6%)	
Voriconazole	4 (14.3%)	44 (19.2%)	
Micafungin	3 (10.7%)	20 (8.7%)	
Itraconazole	3 (10.7%)	18 (7.9%)	
Amphotericin B (liposomes or lipid dispersions)	0	5 (2.2%)	
Two-drugs combination^b^	0	4 (1.7%)	
Treatment duration, mean ± SD	19.0 ± 13.3	16.7 ± 13.3	0.338
Antifungal therapy >5 days, n (%)	8 (27.6)	70 (26.4)	1.000
Time between ICU admission and diagnosis of Candida infection (days), median (Q1,Q3)	11.0 (4.0, 26.0)	10.00 (4.0, 21.0)	0.544

*P<0.05, **P<0.01.

^a^Use of immunoglobulins and/or thymosin α1.

^b^Fluconazole + caspofungin: 2 patients; itraconazole + fluconazole: 1 patient; amphotericin B + caspofungin: 1 patient.

CRCBSI: catheter-related Candida bloodstream infection; NCRCBSI: non-catheter-related Candida bloodstream infections; ICU: intensive care unit; APACHE: acute physiology and chronic health evaluation II; SOFA: sequential organ failure assessment.

### Catheter indwelling between the CRCBSI and NCRCBSI groups

Among CRCBSI patients, 26 were inserted with CVC, and the remaining three had peripheral arterial catheters. Among the NCRCBSI patients, 217 were inserted with CVC. There was no difference in the rate of CVC indwelling between CRCBSI and NCRCBSI patients (*P* = 0.438). CVC puncture was placed in the jugular, subclavian, or femoral vein, without difference between the groups (all *P* > 0.05). Indwelling time of the last catheter before candidemia diagnosis was not different between the groups (all *P* > 0.05) (Table 2).

**Table 2:** **Indwelling and removal of catheters according to CRCBSI and NCRCBSI**

Category		CRCBSI	NCRCBSI	***P***-value
		N = 29	N = 265	
Indwelling central venous catheter, n (%)^a^				0.438
Yes		26 (89.7)	217 (81.9)	
No		3 (10.3)	48 (18.1)	
Catheter indwelling position, n (%)				
Jugular vein		12 (41.4)	107 (40.4)	1.000
Subclavian vein		13 (44.8)	104 (39.2)	0.556
Femoral vein		8 (27.6)	75 (28.3)	1.000
Catheter removal from each position, n (%)^b^				
Jugular vein	A	2 (6.9)	19 (7.2)	0.335
	B	0 (0)	22 (8.3)	
Subclavian vein	A	5 (17.2)	17 (6.4)	0.251
	B	2 (6.9)	29 (10.9)	
Femoral vein	A	2 (6.9)	14 (5.3)	0.960
	B	2 (6.9)	20 (7.5)	
Period of catheter indwelling in the last time (days), median (min, max)				
Jugular vein		10.00 (4.0, 13.0)	8.00 (-1.0, 382.0)	0.827
Subclavian vein		14.00 (1.0, 30.0)	9.00 (-337.0, 63.0)	0.150
Femoral vein		5.00 (1.0, 18.0)	9.00 (-58.0, 369.0)	0.161
Catheter removal after diagnosis, n (%)		24 (82.8)	159 (60.0)	0.016*

* *P* < 0.05.

^a^Includes patients who were catheterized within 2 weeks of the first positive sample but in whom the catheter was removed before diagnosis.

^b^Analysis of catheter removal within 2 weeks before diagnosis. A: catheter was removed at some time in the 2-week period before diagnosis, but a catheter was in place at diagnosis. B: catheter was removed in the 2-week period before diagnosis, and there was no catheter in place at diagnosis.

### Catheter removal between the CRCBSI and NCRCBSI groups

There was no difference between the two groups in the number of CVC removal from each position within two weeks before candidemia diagnosis (all *P* > 0.05). Significantly more catheters were removed in the CRCBSI group after diagnosis (82.8 vs. 60.0%, *P* = 0.016) (Table 2).

### Microbiology

Because of the individual hospital policy and suboptimal storage or handling of isolates, not all isolates from other hospitals were sent to the central laboratory for review and identification, and a total of 237 isolates (21 from CRCBSI patients, and 216 from NCRCBSI patients) were identified in the central laboratory (Table 3). The proportion of *Candida parapsilosis* (33.3%) was higher than that of *Candida albicans* (28.6%) in the CRCBSI group, while the proportion of *Candida albicans* (40.3%) was the highest in the NCRCBSI group. However, there was no difference in the distribution of *Candida* strains between the two groups (*P* = 0.352). The results derived from the different hospitals were consistent with those obtained in the central laboratory. Additional file 1: Table S1 presents the strains distribution across study centers in China.

**Table 3:** ***Candida*** **strains according to CRCBSI and NCRCBSI**

Strain	CRCBSI	NCRCBSI	***P***-value
	N = 21	N = 216	
*Candida* strains isolates, n (%)			0.352
*Candida albicans*	6 (28.6)	87 (40.3)	
*Candida parapsilosis*	7 (33.3)	48 (22.2)	
*Candida tropicalis*	2 (9.5)	37 (17.1)	
*Candida glabrata*	2 (9.5)	25 (11.6)	
Others	4 (19.0)	19 (8.7)	

### Multivariate analysis

Multivariate analysis showed that the SOFA score at candidemia diagnosis was independently associated with CRCBSI (odds ratio = 1.142, 95% confidence interval = 1.049-1.244, *P* = 0.002). Solid tumors (*P* = 0.08), chronic hepatic insufficiency (*P* = 0.82), age (*P* = 0.16) and body weight (*P* = 0.58) were not associated with CRCBSI (Table 4).

**Table 4:** **Multivariate logistic regression analysis for exposure to potential risk factors for CRCBSI in ICU patients**

Variables	Estimate	Standard error	Wald chi-square	Odds ratio estimate	Lower 95% confidence limit	Upper 95% confidence limit	***P***-value
Solid tumors (yes *vs.* other Concomitant disease)	0.344	0.1954	3.1008	1.990	0.925	4.279	0.0783
Chronic hepatic insufficiency (yes *vs.* other concomitant disease)	-0.1596	0.7118	0.0503	0.852	0.211	3.44	0.8226
Age	0.0112	0.00808	1.9336	1.011	0.995	1.027	0.1644
SOFA score at diagnosis	0.1329	0.0436	9.2834	1.142	1.049	1.244	0.0023**
Body weight	-0.00817	0.0149	0.3021	0.992	0.963	1.021	0.5825

**P<0.01

### Antifungal treatment

Twenty-eight CRCBSI patients (28/29, 96.6%) received antifungal treatment, without difference from the NCRCBSI group (229/265, 86.4%; *P* > 0.05) (Table 1). The more frequently used drugs were fluconazole, followed by caspofungin and voriconazole. The course of antifungal therapy was similar between the two groups. More CRCBSI patients received immune enhancement therapy (immunoglobulins and/or thymosin α1) (72.4% *vs.* 38.5%; *P* < 0.001) (Table 1). However, because it only was an observational indicator, the types and doses of immunoglobulins and thymosin α1 were not recorded.

### Treatment outcomes

CRCBSI patients showed a non-significant higher mortality (44.8% *vs.* 36.2%; *P* = 0.419). Trends toward longer ICU stay (median: 34 *vs.* 25 days; *P* = 0.095) and hospitalization (median: 54 *vs.* 39 days; *P* = 0.096) were also observed. CRCBSI patients were more likely to experience microbiological recovery compared with NCRCBSI patients (67.9% *vs.* 50.0%; *P* < 0.001) (Table 5).

**Table 5:** **Treatment outcomes according to CRCBSI/NCRCBSI**

Category	CRCBSI	NCRCBSI	***P***-value
	n = 29	n = 265	
Mortality, n (%)	13 (44.8)	96 (36.2)	0.419
ICU stay period (days), median (Q1,Q3)	34.00 (18.0, 71.0)	25.00 (13.0, 44.0)	0.095
Hospital stays (days), median (Q1,Q3)	54.00 (26.0, 91.0)	39.00 (18.0, 69.5)	0.096
*Candida* elimination, n (%)	19 (67.9)	116 (50.0)	0.001**
Time from positive to negative blood culture (days), median (Q1,Q3)	14.00 (6.0, 24.0)	17.00 (12.0, 26.0)	0.275

**P<0.01

## Discussion

To our knowledge, the China-SCAN study is the largest prospective study of invasive candidiasis in Chinese ICUs, and possibly from anywhere. In addition, it is also one of the first to describe *Candida* catheter-related bloodstream infections in China. The present study aimed to determine the risk factors for catheter-related candidemia in Chinese ICU. Our results showed that CRCBSI incidence in ICU was 0.03%, accounting for 9.86% of all candidemia observed in ICU (29/294), mainly caused by *Candida parapsilosis* in CRCBSI patients (33.3%). Univariate analyses showed that older age and lower body weight were associated with CRCBSI. Multivariate analysis showed that the SOFA score was independently associated with CRCBSI (*P* = 0.002). Catheter removal and immune enhancement therapy were more frequently used in CRCBSI than in NCRCBSI. Results of the present study provide clues for a better identification of CRCBSI patients.

Few studies reported large-scale epidemiological data on CRCBSI. In the present study, we reported a CRCBSI incidence in ICUs of 0.3/1000 patients, which was calculated based on the 96,060 ICU patients reported in the China-SCAN study. In the present study, some patients with NCRCBSI did not have blood sample in venous catheter; therefore, some of these patients might in reality be CRCBSI cases. In addition, some *Candida*-positive patients could have been excluded because of the strict inclusion criteria of the CHINA-SCAN study. Therefore, the real CRCBSI incidence may be higher.

In the present study, the mortality rate from CRCBSI was 44.8%, which was not significantly different from that of NCRCBSI (36.2%). These rates are in agreement with the published global mortality rates of 30-61.8% in hospital-based candidemia studies from western countries [5]-[8].

The CRCBSI and NCRCBSI groups were compared in order to identify risk factors for CRCBSI. Results showed that the two groups were similar in disease, invasive procedures, disease severity score, and the use of antibiotics within the past two weeks. However, in univariate analyses, there were significant differences in age and body weight. These results suggest that risk factors for CRCBSI, other candidemias and invasive candidiasis were similar in most ICU patients, except for those with an older age and lower body weight.

CVC is the most common type of catheter causing CRCBSI [28]. Studies have shown that CVC placement rate in candidemia patients is 80-96.7% [21]. Consistent with these results, the CVC placement rates in patients with CRCBSI and those with NCRCBSI in the present study were above 80% (89.7% and 81.9%), and there was no significant different between the two groups. The CVC placement position and indwelling period were similar in both groups, indicating that the initial placement position of catheter and catheter indwelling time were not the cause of CRCBSI. The catheter removal rate was not different within 2 weeks before diagnosis between the two groups, but was significantly higher after diagnosis in the CRCBSI group (82.8%) compared with the NCRCBSI group (60.0%), in compliance with previous studies and guidelines [18],[29],[30]. A recent study suggested that any delay in catheter removal and initiation of antifungal therapy was associated with increased mortality in CRCBSI patients; however, catheter removal had no impact on mortality of NCRCBSI patients [18]. On the other hand, some studies argued that CVC removal do not affect prognosis of candidemia [31]. The 2012 ESCMID guidelines also pointed out that catheter removal is necessary for candidemia, but that antifungal treatment can be used if catheter removal is impossible [32].

In the present study, multivariate analysis showed that the SOFA score was the only independent variable associated with CRCBSI. We explored a number of factors that have been shown to be associated with candidemia in previous studies, but we did not observe any association between these parameters and CRCBSI. The study by MacDonald et al. [19] showed that hyperalimentation was the only independent risk factor for candidemia in an ICU pediatric population. Another pediatric study showed that CVC, cancer, recent use of vancomycin, and use of agents against anaerobic bacteria were independent factors associated with candidemia [8]. A study showed that independent predictors of biofilm-forming candidemia were the use of CVC, the use of urinary catheters, parenteral alimentation, and diabetes [15]. Finally, a study showed that an inadequate antifungal therapy, infection with biofilm-forming *Candida* species, and APACHE III scores were associated with higher *Candida*-related mortality [16]. However, these studies did not differentiate between CRCBSI and NCRCBSI. In addition, the only risk factor for candidemia that was common to these studies was the use of CVC.

Because there is variability in the resistance of *Candida* strains to storage and transport, some samples could not be tested in the central laboratory, and the exact distribution of the different *Candida* species might have suffered from this bias. However, we observed that CRCBSI was mainly associated with *Candida parapsilosis*, while NCRCBSI was mainly associated with *Candida albicans*, although the distribution of strains between the two groups was not different. This observation was consistent with previous studies on candidemia [6],[25],[33]. *Candida parapsilosis* is more prone to cause CRCBSI, which may be related to its ease of growing in intravenous infusion of high-sugar-based nutrition, to its growing in CVC biofilm that can easily be spread by the hands of medical personnel, and to its long-term survival [33]. Using catheter removal and appropriate antifungal therapy, CRCBSI microbiological clearance rate was significantly higher than that of NCRCBSI (67.9% vs. 50.0%), which was consistent with a previous study [18].

There was no difference in antifungal treatment between CRCBSI and NCRCBSI patients in respect to antifungal treatment. The most commonly used was fluconazole, followed by caspofungin and voriconazole. In the China-SCAN flora and sensitivity analysis, patients with non-*albicans* strains were more susceptible to require a therapy adjustment [10]. Considering the high proportion of *Candida parapsilosis* causing CRCBSI in the present study, we suggest to consider drugs with a higher efficacy against *Candida parapsilosis* in the treatment of CRCBSI.

Although there is no clear indicator of the presence of immunosuppression in the present study, ICU physicians rely on clinical experience to use immunotherapy. More CRCBSI patients received immune enhancement therapy (72.4% *vs.* 38.5%), suggesting that ICU physicians are concerned about CRCBSI-related immune suppression. From the previously identified risk factors in patients with CRCBSI [8],[15]-[19], patients with old age and low body weight might be more prone to develop immunosuppression. Although no immune enhancement therapy is clearly recognized to improve CRCBSI patients' prognosis, immunoglobulins and thymosin α1 were selected as immunotherapy since these drugs have a potential to improve prognosis in sepsis patients [34],[35].

The China-SCAN study suffered from some limitations that also have an impact in the present study. Indeed, the lack of central validation for some samples could lead to an underestimation of the real CRCBSI incidence, as well as for the *Candida* strains causing CRCBSI. However, results based from the central laboratory clearly demonstrated that the proportion of *Candida parapsilosis* was higher in the CRCBSI group, while that of *Candida albicans* was higher in the NCRCBSI group. Differences in therapeutic strategies across the study centers may have contributed to biases in diagnosis, treatments and prognosis. Concerning the present study, the sample size of the CRCBSI group was small. Therefore, results need to be verified in further large-scale studies on CRCBSI.

## Conclusions

In China, CRCBSI was more likely to occur in old patients with low body weight. SOFA score was independently associated with CRCBSI. *Candida parapsilosis* accounted for a high proportion of CRCBSI, but the difference from NCRCBSI was not significant.

## Authors' contributions

BH, JL and HQ designed the study. BH, ZD contributed to the manuscript development. ZD, YK, BZ, WC, BQ, QF and HQ were involved in patient recruitment and served as study investigators. All authors read and approved the final manuscript.

## Additional file

## Electronic supplementary material

Additional file 1: Table S1.: Candida strains by center. (DOCX 26 KB)

Below are the links to the authors’ original submitted files for images.

Authors’ original file for figure 1
