# Supplementary material for: Catheter-related Candidabloodstream infection in intensive care unit patients: a subgroup analysis of the China-SCAN study
Source: BMC Infect Dis. 2014 Nov 13;14:594. doi: 10.1186/s12879-014-0594-0 (PMC4234860; doi:10.1186/s12879-014-0594-0)
Supplement: Supplementary file 1 — Additional file 1: Table S1.: Candida strains by center. (DOCX 26 KB) [file 12879_2014_594_MOESM1_ESM.docx]

**Additional file 1: Table S1.** Candida strains by center

| **Candida strains by center** | **Number of pathogens** |
| --- | --- |
| **Affiliated Hospital of Guiyang medical college, emergency ICU** | **3** |
| *Candida albicans* | 3 |
| **Affiliated Hospital of Guiyang medical college, surgical ICU** | **7** |
| *Candida albicans* | 3 |
| *Candida tropicalis* | 2 |
| *Candida albicans+Candida parapsilosis* | 1 |
| *Candida glabrata* | 1 |
| **Affiliated Hospital of Nantong university** | **7** |
| *Candida albicans* | 5 |
| *Candida parapsilosis* | 1 |
| *Candida tropicalis* | 1 |
| **Beijing Tiantan Hospital affiliated to Capital Medical University** | **3** |
| *Candida albicans* | 2 |
| *Candida tropicalis* | 1 |
| **Beijing Tongren Hospital Capital medical university** | **4** |
| *Candida albicans* | 2 |
| *Candida glabrata* | 1 |
| *Candida tropicalis* | 1 |
| **Chinese PLA General Hospital** | **3** |
| *Candida albicans* | 1 |
| *Candida parapsilosis* | 1 |
| *Candida tropicalis* | 1 |
| **Daping Hospital, Southwest Hospital** | **11** |
| *Candida albicans* | 6 |
| *Candida haemulonii* | 2 |
| *Candida glabrata* | 1 |
| *Candida guilliermondii* | 1 |
| *Candida parapsilosis* | 1 |
| **First Affiliated Hospital of Dalian Medical University** | **2** |
| *Candida albicans* | 1 |
| *Candida parapsilosis* | 1 |
| **First Affiliated Hospital of Kunming Medical University** | **3** |
| *Candida albicans* | 1 |
| *Candida glabrata* | 1 |
| *Candida tropicalis* | 1 |
| **First Affiliated Hospital of Medical College of Xi'an Jiaotong University** | **4** |
| *Candida glabrata* | 2 |
| *Candida albicans* | 1 |
| *Candida parapsilosis* | 1 |
| **General Hospital of Ningxia Medical University** | **5** |
| *Candida albicans* | 3 |
| *Lodderomyceselongisporus* | 1 |
| *Candida tropicalis* | 1 |
| **Guangdong General Hospital** | **10** |
| *Candida parapsilosis* | 6 |
| *Candida guilliermondii* | 2 |
| *Candida glabrata+Candida parapsilosis* | 1 |
| *Candida tropicalis* | 1 |
| **Hebei Medical University Fourth Hospital** | **1** |
| *Candida glabrata* | 1 |
| **Henan Provincial People's Hospital** | **10** |
| *Candida tropicalis* | 4 |
| *Candida parapsilosis* | 3 |
| *Candida albicans* | 2 |
| *Candida glabrata* | 1 |
| **Jiangsu Subei People's Hospital** | **4** |
| *Candida glabrata* | 2 |
| *Candida parapsilosis* | 1 |
| *Candida tropicalis* | 1 |
| **Jinlin Hospital** | **8** |
| *Candida albicans* | 2 |
| *Candida glabrata* | 2 |
| *Wickerhampmycesanomalus(Pichiaanomala,theteleomorphofC.pelliculosa)* | 1 |
| *Candida glabrata+Candida tropicalis* | 1 |
| *Candida parapsilosis* | 1 |
| *Candida tropicalis* | 1 |
| **Ningbo First Hospital** | **6** |
| *Candida parapsilosis* | 3 |
| *Candida albicans* | 2 |
| *Candida glabrata* | 1 |
| **Peking Union Medical College Hospital** | **5** |
| *Candida albicans* | 4 |
| *Candida parapsilosis* | 1 |
| **Qilu Hospital of Shandong University** | **4** |
| *Candida parapsilosis* | 2 |
| *Candida albicans* | 1 |
| *Candida guilliermondii* | 1 |
| **Shandong Provincial Hospital** | **1** |
| *Candida tropicalis* | 1 |
| **Shanghai Changzhen Hospital** | **3** |
| *Candida parapsilosis* | 2 |
| *Candida haemulonii* | 1 |
| **Shengjing Hospital, affiliated to China Medical University** | **12** |
| *Candida parapsilosis* | 5 |
| *Candida albicans* | 4 |
| *Candida glabrata* | 1 |
| *Candida norvegensis* | 1 |
| *Candida tropicalis* | 1 |
| **Southwest Hospital, Southwest Hospital** | **1** |
| *Candida glabrata* | 1 |
| **The 2nd Affiliated Hospital of Harbin Medical University** | **2** |
| *Candida albicans* | 2 |
| **The 2nd Affiliated Hospital of Harbin Medical University** | **1** |
| *Candida parapsilosis* | 1 |
| **The Affiliated Hospital of Medical College Qingdao University** | **2** |
| *Candida tropicalis* | 2 |
| **The First Affiliated Hospital with Nanjing Medical University** | **2** |
| *Candida albicans+Candida glabrata* | 1 |
| *Candida parapsilosis* | 1 |
| **The First Affiliated Hospital of Fujian Medical University** | **7** |
| *Candida albicans* | 2 |
| *Candida parapsilosis* | 2 |
| *Candida albicans+Candida parapsilosis* | 1 |
| *Candida albicans+Candida tropicalis* | 1 |
| *Candida haemulonii* | 1 |
| **The First Affiliated Hospital of Guangxi Medical University** | **4** |
| *Candida parapsilosis* | 2 |
| *Candida albicans* | 1 |
| *Candida tropicalis* | 1 |
| **The First Affiliated Hospital of Guangxi Medical University, Surgery** | **3** |
| *Candida albicans* | 2 |
| *Candida tropicalis* | 1 |
| **The First Affiliated Hospital of Harbin Medical University** | **5** |
| *Yarrowialipolytica(anamorph:candidalipolytica)* | 2 |
| *Candidaernobii(torulopsisernobii)* | 1 |
| *Candida albicans* | 1 |
| *Candida tropicalis* | 1 |
| **The First Affiliated Hospital of Lanzhou University** | **6** |
| *Candida glabrata* | 3 |
| *Candida albicans* | 2 |
| *Candida parapsilosis* | 1 |
| **The First Affiliated Hospital of Medical School of Zhejiang University** | **11** |
| *Candida albicans* | 4 |
| *Candida parapsilosis* | 3 |
| *Candida tropicalis* | 3 |
| *Candida glabrata* | 1 |
| **The first affiliated hospital of Soochow University** | **2** |
| *Candida albicans* | 1 |
| *Candida glabrata* | 1 |
| **The First Affiliated Hospital of Soochow University, Emergency ICU** | **5** |
| *Candida albicans* | 3 |
| *Candida parapsilosis* | 1 |
| *Candida tropicalis* | 1 |
| **The First Affiliated Hospital of Wenzhou Medical College** | **4** |
| *Candida albicans* | 2 |
| *Candida glabrata* | 1 |
| *Candida tropicalis* | 1 |
| **The First Affiliated Hospital of Zhengzhou university** | **1** |
| *Candida albicans* | 1 |
| **The first affiliated hospital SUN YAT-SEN university** | **5** |
| *Candida tropicalis* | 3 |
| *Candida albicans* | 1 |
| *Candida parapsilosis* | 1 |
| **The First Teaching Hospital of Xinjiang Medical University** | **2** |
| *Candida albicans* | 1 |
| *Candida tropicalis* | 1 |
| **The Second Affiliated Hospital of Soochow University** | **6** |
| *Candida albicans* | 4 |
| *Candida parapsilosis* | 2 |
| **The Second Affiliated Hospital of Zhejiang University School of Medicine** | **13** |
| *Candida albicans* | 7 |
| *Candida glabrata* | 2 |
| *Candida parapsilosis* | 2 |
| *Candida tropicalis* | 2 |
| **The Second Hospital of Jilin university** | **1** |
| *Candida albicans* | 1 |
| **Tianjin First Center Hospital** | **2** |
| *Candida parapsilosis* | 1 |
| *Candida krusei* | 1 |
| **Tianjin Third Central Hospital** | **7** |
| *Candida parapsilosis* | 4 |
| *Candida glabrata* | 2 |
| *Candida albicans* | 1 |
| **Tongji Hospital, Tongji Medical College, Huazhong University of Science and Technology** | **1** |
| *Candida parapsilosis* | 1 |
| **Wuhan Union Hospital, Tongji Medical College, Huazhong University of Science and Technology** | **1** |
| *Candida parapsilosis* | 1 |
| **Wuxi People's Hospital** | **2** |
| *Candida albicans* | 1 |
| *Candida glabrata* | 1 |
| **Xiangya Hospital Central-South University** | **3** |
| *Candida albicans* | 2 |
| *Candida tropicalis* | 1 |
| **Xuzhou Central Hospital** | **6** |
| *Candida albicans* | 3 |
| *Candida tropicalis* | 2 |
| *CandidapelliculosaAPI-6406074(teleomorph:Pichiaanomala)* | 1 |
| **Zhejiang Hospital** | **1** |
| *Candida albicans* | 1 |
| **Zhongda Hospital, Southeast University** | **5** |
| *Pichaanomala(theteleomorphof C.pelliculosa)* | 1 |
| *Candida albicans* | 1 |
| *Candida glabrata* | 1 |
| *Candida parapsilosis* | 1 |
| *Candida tropicalis* | 1 |
| **Zhongnan Hospital of Wuhan University** | **3** |
| *Candida parapsilosis* | 2 |
| *Candida albicans* | 1 |
| **Zhongshan Hospital Fudan University** | **7** |
| *Candida albicans* | 5 |
| *Candida tropicalis* | 2 |
| **Total** | **237** |
