# Supplementary figures and images for: Catheter-related Candidabloodstream infection in intensive care unit patients: a subgroup analysis of the China-SCAN study
Source: BMC Infect Dis. 2014 Nov 13;14:594. doi: 10.1186/s12879-014-0594-0 (PMC4234860; doi:10.1186/s12879-014-0594-0)

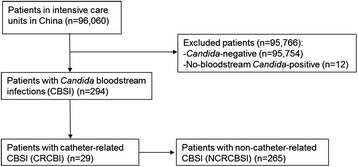

Supplement: Supplementary file 2 — Authors’ original file for figure 1 [file 12879_2014_594_MOESM2_ESM.gif]
